# Sustainability in Abdominal Wall Reconstruction: An Eco-Audit of the Abdominal Wall Reconstruction Pathway

**DOI:** 10.1097/AS9.0000000000000576

**Published:** 2025-05-15

**Authors:** Zahra Ahmed, Alexander Zargaran, Andia Soltani, Sara Sousi, David Zargaran, Yazan Al-Ajam, Shadi Ghali, Afshin Mosahebi

**Affiliations:** From the * University College London, London, United Kingdom!; † Department of Plastic and Reconstructive Surgery, Royal Free NHS Foundation Trust, London, United Kingdom.

**Keywords:** abdominal wall reconstruction, carbon footprint, healthcare sustainability, lifecycle assessment, process mapping, sustainability

## Abstract

**Objective:**

This study aimed to perform process mapping and life cycle assessment of patients who underwent abdominal wall reconstruction to identify actionable carbon hotspots, decrease emissions, and increase sustainability.

**Background:**

Abdominal wall reconstruction is a procedure requiring input from multiple specialities and is often performed on complex multimorbid patients requiring a high level of care, the environmental impact of which has yet to be explored.

**Methods:**

A retrospective study was conducted on 30 patients who underwent abdominal wall reconstruction at a single center. Process mapping and life-cycle analyses were performed for surgical and inpatient stay, as well as preoperative and outpatient evaluation including facilities, consumables, medical gases, equipment, food and linen, and travel. Estimates for carbon dioxide emissions were generated for each stage, with variability considered, as well as potential areas for savings.

**Results:**

This study estimated the carbon footprint of a patient undergoing abdominal wall reconstruction surgery to be approximately 420.56 kgCO_2_eq. Inpatient stay had the highest overall contribution to the carbon footprint (316.9 kgCO_2_eq., 75.4% pathway emissions). From non-inpatient analysis, patient travel was the predominant source of carbon emissions (51.8 kgCO_2_eq, 50.0%) followed by the production and transport of equipment and building electricity, gas, oil, and water usage.

**Conclusions:**

This is the first study to estimate the carbon footprint of a surgical pathway for complex patients, using abdominal wall reconstruction as an example. Strategies to combat the impact of carbon emissions and increase sustainability included greater implementation of enhanced recovery after surgery protocols to reduce inpatient stay, improved accuracy of waste segregation, and continued use of total intravenous anesthesia.

## INTRODUCTION

Climate change is a considerable threat to humanity with both severe financial and health implications.^1,2^ The World Health Organization predicted that climate change will result in 250,000 further deaths annually between 2030 and 2050 and by 2030; The estimated direct damage costs to health were projected to reach up to 4 billion US Dollars annually. As defined in the Paris Agreement, limiting global warming to less than 1.5°C is paramount. This objective demanded a 45% drop in emissions by 2030 and a shift to a carbon net-zero world by 2050, representing a significant challenge humankind must tackle.^3^

Healthcare systems place significant pressure on the environment through the production of greenhouse gas emissions, hazardous waste, and wastewater.^4^ The healthcare division generates 4·4% of greenhouse gas emissions globally.^5–7^ Surgery is one of the most resource-consuming sectors (areas) within healthcare, with operating theaters using up to 6 times more energy than other hospital areas, and generating 30% hospital waste.^8^

In response to the increasingly urgent threat to health from climate change, the National Health Service (NHS) in the United Kingdom (UK) became the first healthcare system in the world to pledge to achieve carbon net zero.^9^

While efforts had been made locally within NHS trust to decrease emissions, the British Medical Association reported a halt in progress toward the NHS goal of net zero by 2040 and reported that carbon monitoring by all trusts was crucial in assessing progress and reaching goals.^10–12^ Life Cycle Assessment can evaluate a product’s or process’ effect on the environment, enabling quantification of energy and resource usage.^13^ Data generated and integrated process mapping can transform the patient pathway to enhance the efficiency of healthcare management.^14^ Glynou et al^15^ identified solutions to evaluate sustainability for surgical processes and Ahmed et al^16^ demonstrated these solutions within reconstructive breast surgery.

Abdominal wall reconstruction (AWR) is an umbrella term to cover the techniques used to reconstruct, restore structure, function, and cosmesis of the abdominal wall. AWR is a versatile surgical procedure with different indications including after hernia repair and following extreme weight loss.^17^ The precise description of a functional abdominal wall has not been universally defined; This study included patients undergoing AWR for various indications including hernia repair, massive weight loss, and trauma.

Reconstructive abdominal surgery requires input from a multidisciplinary team and patients are often multimorbid. However, the environmental impact of the pathway has yet to be evaluated. Therefore, the aim of this study was to process map and perform a thorough life cycle assessment of the AWR pathway to quantify its carbon footprint and determine carbon hotspots that could indicate areas for reduction, ultimately leading to increased sustainability within AWR.

## METHODS

### Study Design and Population

**FIGURE 1.**
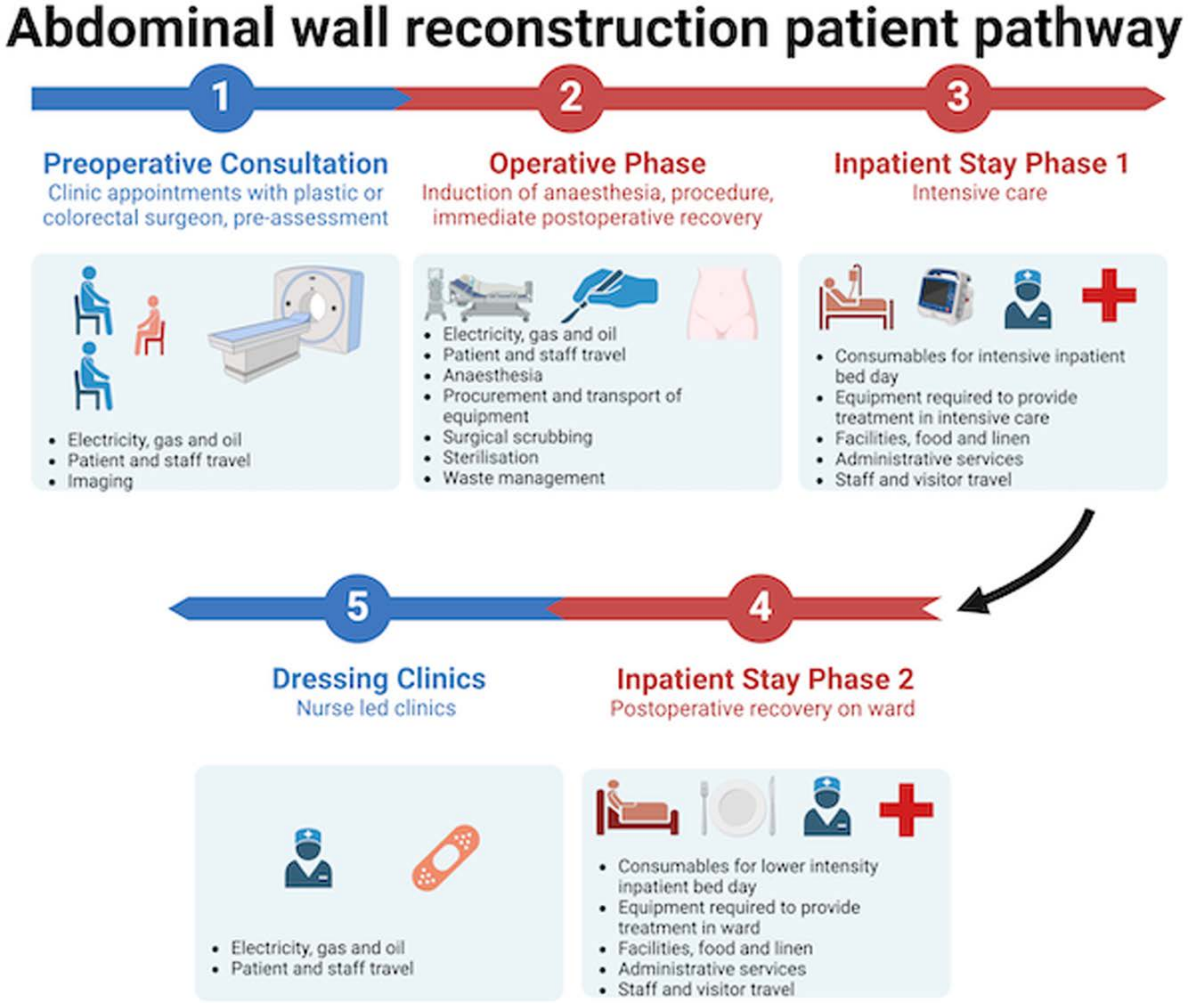
Abdominal wall reconstruction pathway.

A retrospective service evaluation was performed using data collected from all patients who underwent AWR from March 24, 2023, to July 7, 2024, in a single plastic surgery department at a large teaching hospital in London. This study was registered as an audit at our trust. Process mapping was performed (Fig. 1), with inclusion criteria limited to AWR without additional simultaneous procedures performed. Life cycle assessment was performed in accordance with the International Standards Organization (ISO) 14040 guidelines.^18^

Data were categorized into 5 time points, including both outpatient and inpatient settings. A hybrid approach was adopted using a bottom–up approach (analyzing each process and material within a product’s life cycle) in all stages except for the inpatient stay stages, which were calculated using a top–down approach (starting with broader data, such as industry averages or national statistics to estimate environmental impact at a higher level).

### Data Collection

Following audit registration at our trust, direct data collection for pathway stages calculated using a bottom–up approach (non-inpatient stages) was collected through Electronic Patient Records, intraoperative raw data collection, surveys, and communication with hospital departments, including procurement, the central sterilization unit and estates, as well as contact with external suppliers. Where raw data were unavailable, reasonable estimates were made using existing literature as benchmarks. Table 1 details data variables and sources.

**TABLE 1.** Bottom–Up Methodology Used to Collect Data Variables

Variable	Methodology
Patient variables	
Postcode Indication for AWR Procedure details	Direct data collection from Electronic Patient Records
Consultation period variables	
Number of in-person consultations before AWR and number of postoperative dressing clinics Any imaging undertaken	Direct data collection from Electronic Patient Records
Number and type of staff present Clinic durations	Through observation
Staff travel	Staff travel surveys
Electricity, gas, oil, and water used	Annual hospital consumption provided by estates
Operative period variables	
Time in anesthetic room, operating theater, and postoperative recovery area Number and type of staff present	Direct data collection from the World Health Organization Intraoperative Record, available on Electronic Patient Records
Equipment used	Direct data collection from the World Health Organization Intraoperative Record and from data sheets inside instrument packs, available on Electronic Patient Records, through observation and discussion with consultant anesthetists and scrub nurses.
Anesthetic agent used	Direct data collection from anesthesia records, available on Electronic Patient Records and discussion with consultant anesthetists
Staff travel	Staff travel surveys
Electricity, gas, oil, and water used	Annual hospital consumption provided by estates
Room sizes	Provided upon request by estates
Equipment transport	Suppliers for equipment and postcodes provided by estates Weight of equipment taken from supplier information sheets
Sterilization water and electricity consumption	Provided upon request by the central sterilization department
Weight of waste	Raw data collection through physical weighing of all constituent bags for different waste streams Weight of sharps bin before and after procedure Raw data obtained from second stage and scaled accordingly by number of suture needles for sharps, number of swabs used for noninfectious offensive waste and number of staff for dry mixed recyclable waste.
Waste management	Provided upon request by the waste management department

Data were stratified by time frame and categorized by sector.

### Carbon Footprint Calculations

For the non-inpatient analysis, the carbon footprint for each sector of the patient journey on the process map was calculated and summed in the units of carbon dioxide equivalents (CO_2_eq.) using multiplication factors derived from sources such as government reports, previous studies, and online resources (Supplementary Table 1, see https://links.lww.com/AOSO/A502). For patients with multiple appointments on the same day, emissions from travel were calculated based on 1 appointment, but emissions from electricity, gas, and oil were calculated based on 2 appointments’ usage.

For the inpatient stay, estimates were based on NHS England’s guidance on appraising inpatient sustainability.^19^

### Boundary Setting

This study followed patients from their first encounter with plastic surgery for AWR up until their final dressing clinic. A hybrid approach was adopted using top–down calculations only where bottom–up calculations were not feasible; In this study, a top–down approach was utilized for inpatient stay due to the variable nature of inpatient stays, including medications received, personnel involved, and catering. Different estimates of the carbon footprint were made depending on the level of care patients required postoperatively. Follow-up beyond dressing clinics was not included due to the heterogeneous nature of additional interventions or procedures performed.

## RESULTS

### Patient Characteristics

There were 30 patients who underwent AWR between March 24, 2023, to July 7, 2024, at our center (Table 2). The most common indication for AWR was hernias (53.3%, N = 16) including incisional hernia with gangrene, supraumbilical, inguinal, and other complex abdominal wall hernias. The second most common indication for AWR was following excess panniculus of abdomen (23.3%, N = 7). Further indications for AWR included abdominal wall injury, open abdominal wounds, tumors, changes in skin lesions, and hypertrophic scars. Differences were observed based on indication for AWR and are detailed in Supplementary Table 2, see https://links.lww.com/AOSO/A503. Most patients had an anteroposterior computerized tomography scan with contrast preoperatively and 5 in-person consultation appointments with plastic surgeons, colorectal surgeons, anesthetists and psychologists, requiring travel to our center an average of 4 times due to some appointments being scheduled on the same day.

**TABLE 2.** Average Variables Used to Calculate CO_2_eq for 23 Patients Undergoing Abdominal Wall Reconstruction

Variable	N (%) or Mean (SD)
Total number of patients	30 (100)
Indication for AWR	
Hernia	16 (53.33)
Excess panniculus of abdomen	7 (23.33)
Abdominal wall injury/open abdominal wound	3 (10)
Tumor	2 (6.67)
Hypertrophic scar	1 (3.33)
Change in skin lesion	1 (3.33)
Most common form of preoperative imaging	AP CT with contrast
Average no. in-person consultations before first stage	5 (2.77)
Average no. days spent on ward	7 (8.64)
Intensive care	1 (2.29)
Ward-based care	6 (7.09)
Average no. postoperative dressing clinics	3 (4.00)
Average distance from patient postcode to hospital (km)	19.11 (22.17)
Average timings for operative phase (minutes) and % operative phase	
Total	367 (95.85) (100)
Anesthetic room	26 (15.62) (4.36)
Theater	198 (71.50) (53.95)
Recovery area	143 (106.26) 38.96)
Most common method of anesthesia	TIVA with propofol + remifentanil
Average no. staff in first operative phase	
Total	10 (1.34)
Surgeons	2 (1.06)
Theater practitioners	4 (0.73)
Anesthetists	2 (1.48)
Healthcare assistants	1 (0.74)
Recovery nurse	1 (0.32)
Average distance from staff postcode to hospital (km)	12.77 (11.19)
Most common method of staff transport	
Surgeon	Overground/underground
Theater practitioner	Train
Anesthetist	Cycle
Healthcare assistants	Walk
Recovery nurse	Overground/underground
Average distance from equipment supplier postcode to hospital (km)	206.30 (147.48)
Weight of waste (kg) and % total waste weight	
Total	8.05 (100)
Sharps	0.6 (7.45)
Noninfectious offensive waste	3.74 (46.46)
Dry mixed recyclable waste	3.71 (46.09)

AP indicates anteroposterior; CT, computerized tomography; SD, standard deviation.

Postoperatively, patients spent an average of 1 day in intensive care and 6 days as inpatients on the ward. Following discharge, patients had an average of 2 outpatient nurse-led dressing clinics, traveling an average of 22.8 km each way to our center.

During the operative phase, patients spent almost as much time in the immediate postoperative recovery area as in theater (197 minutes in theater and 155 minutes in the recovery suite). The most common method of anesthesia was total intravenous anesthesia (TIVA) with propofol and remifentanil. An average of 10 staff per patient were involved in the operative phase, traveling an average distance of 12.8 km to work using different methods of transport. Different suppliers were used for equipment procurement and were transported an average of 206.3 km from manufacturer to our center. There was a total of 8.05 kg of waste produced from the AWR procedure. Waste was divided into 3 streams: sharps, noninfectious offensive, and dry mixed recyclable waste. The majority of waste was almost equally divided between noninfectious offensive and dry mixed recyclable waste (46.5% and 46.1% total weight, respectively).

There were 11 surgeons who performed AWR. As this study was performed at a teaching hospital, both trainees (registrar) and consultants were operating surgeons. Two consultant surgeons were responsible for almost 3 quarters of the patients in this study (N = 22, 73.3%) and oversaw many of the trainees. There was little variability in operating time between surgeons.

### Overall Carbon Footprint

**FIGURE 2.**
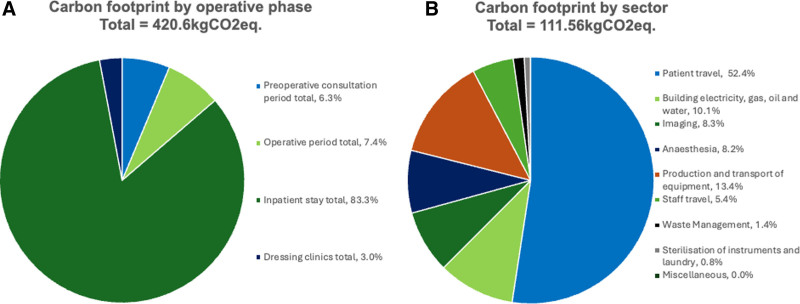
A, Pie chart showing the proportion of carbon emissions from each phase of the abdominal wall reconstruction patient pathway. B, Pie chart showing the proportion of carbon emissions from each sector involved in the bottom–up approach calculating emissions from the abdominal wall reconstruction patient pathway.

**FIGURE 3.**
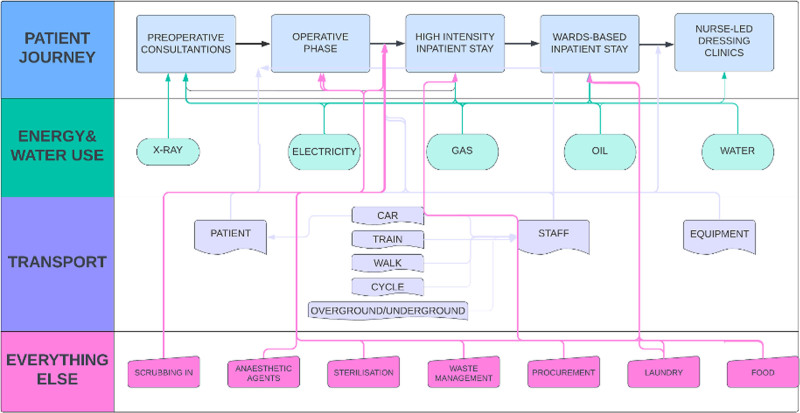
Contributing factors to overall emissions in the abdominal wall reconstruction pathway.

The total carbon footprint for a patient undergoing AWR, from first encounter with the plastic surgery department to their final dressing clinic postoperatively, was calculated to be 420.6 kgCO_2_eq., with multiple contributing factors (Fig. 3). Inpatient stay was by far the greatest contributor to overall carbon emissions during the AWR pathway (316.9 kgCO_2_eq., 75.4% overall emissions, Table 3 and Fig. 2A).

**TABLE 3.** Carbon Dioxide Equivalents (kgCO_2_eq.) for Each Sector in Each Operative Period

	KgCO_2_eq. (% Overall Emissions)
Total	669.56 (100)
Preoperative consultation period total	42.19 (6.30)
Operative period total	49.51 (7.39)
Inpatient stay total	558.0 (83.34)
Dressing clinics total	19.86 (2.97)
Preoperative consultation total	42.19 (6.30)
Electricity, gas, and oil	0.20 (0.03)
Patient travel	32.48 (4.85)
Staff travel	0.31 (0.05)
Imaging	9.20 (1.37)
Operative period total	49.51 (7.39)
Electricity, gas, oil, and water	10.91 (1.63)
Patient travel	6.50 (0.97)
Staff travel	5.50 (0.82)
Anesthesia	9.15 (1.36)
Production of equipment	13.57 (2.03)
Transport of equipment	1.34 (0.2)
Surgical scrubbing	0.05 (0.01)
Sterilization of instruments	0.38 (0.05)
Waste management	1.60 (0.24)
Laundry of gowns	0.51 (0.08)
Inpatient stay^*^	558 (83.34)
Intensive care	179 (26.73)
Ward-based care	379 (56.61)
Dressing clinics after first stage	19.86 (2.97)
Electricity, gas, and oil	0.12 (0.02)
Patient travel	19.49 (2.91)
Staff travel	0.25 (0.04)

* Calculated using a top–down approach from NHS England’s guidance on appraising sustainability inpatient bed day module.

### Consultation Period Contributors

The preoperative consultation period contributed 40.7 kgCO_2_eq. to the carbon footprint (equivalent to driving 238.8 km in a petrol car). This was primarily due to emissions from patient travel, followed by imaging (76.2% and 22.6% consultation period emissions respectively). The remaining emissions from the consultation period were from electricity, gas, and oil building usage and staff travel (0.5 kgCO_2_eq.).

### Operative Period Contributors

The operative period was the second greatest contributor to emissions across the AWR pathway, totaling 49.7 kgCO_2_eq. (11.8%), equivalent to driving 291.6 km in a petrol car. Equipment was responsible for over a quarter of emissions in the operative phase (13.2 kgCO_2_eq., 26.6% operative period emissions), followed by electricity, gas, oil, and water usage and anesthesia (22.7% and 16.6% operative emissions, respectively). Patient and staff travel combined produced 13.1 kgCO_2_eq. (26.3% operative phase emissions). The transport of equipment, surgical scrubbing, sterilization of instruments, waste management, and laundry made up the remaining 7.8% of emissions (3.9 kgCO_2_eq.).

### Inpatient Stay Contributors

Inpatient stay was calculated using a top–down approach and contributed a huge proportion to total AWR pathway emissions (316.9 kgCO_2_eq., 75.4% overall pathway emissions, equivalent to driving 1858.8 km in a petrol car). Per-day emissions were greater for intensive care bed days than for ward-based care (89.5 vs 37.9 kgCO_2_eq. for intensive care and ward-based care, respectively). However, since patients spent more days on lower level care wards (1 day in intensive care vs 6 days in ward-based care), the overall emissions throughout the AWR pathway from ward-based care were 2 and a half times greater than those from intensive care (227.4 vs 89.5 kgCO_2_ emitted for ward-based vs intensive care).

### Dressing Clinic Contributors

Nurse-led patient dressing clinics contributed 13.2 kgCO_2_eq. to the overall pathway (3.15% overall pathway and 12.8% non-inpatient emissions), the equivalent of driving 77.6 km in a petrol car. Emissions were predominantly attributable to patient travel (12.99 kgCO_2_eq., 98.4% dressing clinic emissions). Remaining emissions were from staff travel and building electricity, gas, and oil usage.

### Sector Analysis

From non-inpatient analysis, after summing emissions data across all aspects of the AWR pathway, patient travel was the greatest source of carbon emissions (51.8 kgCO_2_eq., 50.0%, Table 4 and Fig. 2B). This was followed by the production and transport of equipment and building electricity, gas, oil, and water usage (14.6 and 11.6 kgCO_2_eq., respectively). Preoperative imaging contributed more than intraoperative anesthesia (9.20 and 8.24 kgCO_2_eq., respectively). Remaining emissions in decreasing order of carbon contributions were from staff travel, waste management, sterilization of instruments, and laundry.

**TABLE 4.** Carbon Dioxide Equivalents (kgCO_2_eq.) for Each Sector Calculated Using Bottom–Up Approach

	KgCO_2_eq. (%)
Total	111.56 (100)
Patient travel	58.47 (52.41)
Building electricity, gas, oil, and water	11.23 (10.07)
Imaging	9.20 (8.25)
Anesthesia	9.15 (8.20)
Production and transport of equipment	14.91 (13.37)
Staff travel	6.06 (5.43)
Waste management	1.60 (1.43)
Sterilization of instruments and laundry	0.89 (0.80)
Miscellaneous	0.05 (0.04)

Excludes inpatient stay. Miscellaneous includes surgical scrubbing.

## DISCUSSION

The climate crisis has been described as this century’s biggest threat to human health.^20,21^ Healthcare is increasingly responsible for global emissions, and without change, emissions are predicted to more than triple by 2050.^22^

This study quantified the carbon footprint of AWR, providing a comprehensive assessment across the patient journey. Several factors contributing to the carbon footprint have been identified (Fig. 3). This study mapped the patient pathway through its various interactions with healthcare services and teams and estimated the total carbon footprint for a patient undergoing AWR to be 420.56 kg CO_2_eq. This was equivalent to driving a petrol car for 1676 km or flying 4673 km, approximately the distance from London to Kuwait.^23^

Patients undergoing AWR are often complex with multiple comorbidities, necessitating a high level of care, input from multiple specialties, meticulous preoperative planning, and close follow-up. This study found that patients had an average of 5 in-person consultations before surgery with a range of healthcare professionals including surgeons, anesthetists, and psychologists. Consequently, the consultation period contributed 40.7 kgCO_2_eq., accounting for 9.7% of overall pathway emissions, and produced almost as many emissions as from the operative phase, which was traditionally thought to be the most resource- and emission-heavy phase of the operative pathway. MacNeill et al^24^ reported that operating theaters were up to 6 times more energy-intense than the hospital as a whole, highlighting the significance of the emissions from the preoperative consultation phase in this study.

Strategies to reduce emissions in the preoperative phase had started to be implemented. Some patients with multiple preoperative appointments were scheduled to see different healthcare professionals on the same day, reducing patient travel and, hence, emissions. However, this was not always this case and should have been considered more when scheduling patient appointments. Further potential solutions to reduce the number of clinic appointments and maximize efficiency would be to run more multidisciplinary clinics. Multidisciplinary care within plastic surgery was found to reduce length of stay, complication rate, and resource consumption and provide financial cost savings.^25^ An increased utilization of multidisciplinary clinics would provide benefits to all parties involved in the AWR pathway, including the environment.

Emissions from the procurement and transport of equipment were responsible for almost a third of total operative phase emissions during the AWR pathway (13.2 kgCO_2_eq., 26.6% operative phase emissions). Due to the multidisciplinary nature of AWR, 13 different types of equipment trays were utilized across the 30 patients in this study, with no patient having the same standard set of equipment used. It could be suggested that much of the equipment opened was not used. Stockert et al^26^ reported that, within plastic surgery, 17% of instrument trays had minimal use, leading to excess carbon emissions from procurement, transport, sterilization or waste management and additional financial costs. Consideration should be taken into creating standardized AWR packs depending on the indication for surgery to reduce emissions. Policymakers should be guided by frameworks such as that layed out by Vollebergh et al^27^ to aid the low-carbon transition.

In this study, an average of 8.05 kg waste was produced per AWR, most of which was almost equally attributable to noninfectious offensive waste or dry mixed recyclable waste (46.5% and 46.1%, respectively). In 2021/2022, only 16% of the generated 487,000 tonnes of direct waste produced by the NHS was recycled.^28^ This study showed that a greater proportion of waste was being recycled raising 2 hypotheses: either operative staff, knowing somebody would weigh the waste at the end of the procedure, put more conscious thought into whether or not something could be recycled, or there was incorrect waste segregation due to insufficient staff education. If the former was true, initiatives to encourage recycling should be implemented, such as posters around theaters and automated reminders during checklists such as the instrument count list. Further guidance should be available on whether items are recyclable through an easy-access electronic inventory.

Education on correct waste segregation remains pivotal; In the United States, Plezia et al^29^ reported that only 23% of certified registered nurse anesthetists, anesthetists, and anesthesia technicians had received formal waste segregation training. Within the United Kingdom, waste training often forms part of the mandatory training when starting at a new hospital trust; however, these are often not interactive or reviewed regularly. In-person interactive refresher sessions with real-life examples operative staff will be faced with should be given more frequently to reduce incorrect waste segregation and maximize the amount of waste that is correctly recycled. Furthermore, increased correct waste segregation could be monitored through mandatory weighing of waste and documentation to track progress.

Postoperative inpatient emissions accounted for the vast majority of emissions in the AWR pathway (316.9 kgCO_2_eq., 75.4%). Patients in this study spent an average of 2 days in intensive care immediately postoperatively, followed by an average of 10 days on lower intensity wards. The additional 8 days spent in ward-based care compared with intensive care resulted in an overall additional 137.9 kgCO_2_eq. of emissions. This highlights the need to minimize the duration of inpatient stay postoperatively. Enhanced recovery after surgery (ERAS) was first described in 1997 by Kehlet et al as a model of care centering around the reduction in length of stay (LOS) to lower complications rate, promote early recovery, reduce financial cost, and improve patient outcomes.^30–33^ A systematic review and meta-analysis by Lode et al^34^ found that implementation of ERAS protocols in AWR resulted in a reduction in LOS without increasing complications. Enhanced recovery programs can offer an additional advantage of reducing the carbon footprint of procedures by decreasing in-hospital consumption, with a reduction in LOS and reduced usage of equipment such as drains. Therefore, wider implementation should be encouraged.^35^

Patient travel was the largest contributor to emissions in non-inpatient analysis (50.0%). Postoperatively, the majority of emissions from nurse-led dressing clinics were attributable to patient travel (13.0 kgCO_2_eq., 98.1% dressing clinic emissions). Patients traveled an average of 12.77 km each way for an average of 2 dressing clinics at our center. Potential solutions include scheduling dressing clinic appointments with nurses at community practices where possible, which are often closer to patients’ homes. From non-inpatient analysis, building energy usage and anesthetic gases contributed 19.8 kgCO_2_eq., less than half of the emissions from patient travel. A systematic review across multiple specialities by de’Angelis et al^36^ reported anesthetic gases and energy usage to be the greatest contributors to the carbon footprint of various surgical procedures. In this study, the most common method of anesthesia was TIVA, which was shown to yield a greater than 20-fold reduction in greenhouse gas emissions than the traditional use of anesthetic gases.^37^ The lesser contribution of anesthetics to the overall carbon footprint in this study should encourage the continued use of TIVA in AWR.

As with any study of this kind, there were inevitable methodological constraints and limitations. This was a retrospective study in line with ISO 14040, which used bottom–up calculations where possible. However, a top–down approach was necessary for calculating emissions from inpatient stay, due to significant variability not captured by electronic records. There was heterogeneity in indications for AWR, but to increase the reliability of the findings, it was deemed necessary to include all indications, and thus, the key takeaways from this study should focus on the opportunities for pathway remodeling and carbon hotspot reduction rather than definitive values and should be used as a guide to identify carbon hotspots where efforts can be focused to reduce emissions in all centers even those with different protocols and in different countries. Furthermore, there was variability in equipment and instrument trays used; One “P1 Plastics Tray” was utilized in almost all cases in addition to 1 other tray depending on the indication for AWR. Thus, in this study, emissions for tray production, transport, and sterilization were based on the assumption of using 2 P1 trays. Mesh was placed in 65% of patients but there was heterogeneity in the use of biologic and synthetic mesh; hence, it was not included in emission calculations. While these are limitations, we have made a trade-off to include more patients to increase the strength of other findings in process mapping and patient factors at the cost of increased equipment variability which we have been forced to average. Sensitivity analysis was not performed but should be incorporated into future studies to minimize the impact of variability.

Despite these limitations, the study provides a robust framework for understanding and addressing the carbon footprint of AWR, an area previously focused on more routine operations involving single specialities such as cataracts and knee arthroplasty^38,39^ Parker et al^40^ reported that 71% of patients undergoing AWR have 1 or more comorbidities. The overall incidence of multimorbidity is growing globally, and it has been estimated that currently over one-third of adults suffer multiple chronic conditions.^41,42^ It is, therefore, vital that we understand the environmental impact of complex multimorbid patients undergoing surgery, and this study demonstrated that using AWR as an example.

This study followed patients from first consultation with plastic surgery in the preoperative phase until their final dressing clinic at our center. This study demonstrates transferrable findings that can be implemented into other pathways to reduce emissions in pursuit of net zero. Further studies should expand on this work to look at any revisional procedures and continue to look at other complex patient pathways to advise change to reduce carbon emissions and increase sustainability.

## CONCLUSION

This study estimated the carbon footprint of a patient undergoing AWR, to be approximately 420.56 kgCO_2_eq. through process mapping the pathway for patients, equipment, land, and staff. Strategies to mitigate the impact of carbon emissions focused on reducing postoperative inpatient stay and increasing the use of ERAS protocols. Further considerations to increase sustainability included correct waste segregation and minimizing the opening of unused instruments. This is the first study to process map and carbon footprint surgical pathway for complex and multimorbid patients, and further work should be conducted for other complex patient pathways.

## Supplementary Material
