# Supplementary material for: Sustainability in Abdominal Wall Reconstruction: An Eco-Audit of the Abdominal Wall Reconstruction Pathway
Source: Ann Surg Open. 2025 May 15;6(2):e576. doi: 10.1097/AS9.0000000000000576 (PMC12185097; doi:10.1097/AS9.0000000000000576)
Supplement: Supplementary file 1 [file as9-6-e576-s001.pdf]

Variable	Methodology and source
Electricity, gas, oil and water consumption	Number of minutes spent in relevant clinical area*proportion of hospital occupied by area*total hospital consumption in one minute (in kWh for electricity, gas and oil and m^3 for water)*conversion factor from government report(1)<br><br>Electricity, gas and oil calculated for time in clinic, anaesthetic room, theatre and postoperative recovery area.<br><br>Water supply was considered negligible apart from in the operative period.<br><br>Operative usage is assumed to be a resource intensive setting so all usage values were doubled.
Patient travel	Average distance from patient postcode to our hospital by land transport (km) calculated using the website free map tools(2)*conversion factor for car transport(3)<br><br>Return journeys calculated for all journeys prior to the day of surgery.
Staff transport	Average distance from staff postcode to our hospital by land transport (km) calculated using the website free map tools(2)*conversion factor for most populous type of transport for that type of staff member(3). Divided by the number of patients responsible for that day.<br><br>Return journeys calculated for all journeys.
Preoperative imaging	Values taken from study by McAlister et.al(4)
Anaesthesia	TIVA emissions calculated for average weight of patients and average operative time (5).
Production and usage of anaesthetic equipment	Lifecycle assessments provided by external supplier (Supplementary File 1)
Production of surgical equipment and scrubs	Values taken from study by Rizan et.al (6)<br>Mesh excluded due to significant heterogeneity in mesh type.
Equipment transport	Average distance from supplier postcode to our hospital by land transport (miles) calculated using the website free map tools(2)*total equipment weight (ton)* average freight truck emissions per ton-mile(7)
Sterilisation of reusable instruments	Electricity(in kWh) and water(in m^3) consumption for the sterilisation of one instrument tray was provided by the Central Sterilisation Unit.<br><br>Electricity/water consumption*average number of instrument trays used per operation*conversion factor from government report(1)
Waste management	Weight of waste stream (non-infectious offensive , dry mixed recyclable or sharps)*kg CO2 per kg waste in each respective stream for study by Rizan.et al(8)
Surgical scrubbing	Number of minutes spent scrubbing(9)*water used in one scrub(m^3)(9)*total hospital water consumption in one minute (m^3)*conversion factor from government report(1)* average number of scrubs per procedure
Laundry of scrubs	Presumed one load of washing per procedure laundered at 60 degrees(10), kgCO2eq. from Shahmohammadi et.al(11)

Supplementary Table 1: Methodology used to calculate carbon dioxide equivalents (kgCO2eq.) for each sector. Abbreviations: AWR- Abdominal Wall Reconstruction, TIVA- Total Intravenous Anaesthetic

1. GOV.UK. Conversion factors 2023: condensed set (for most users) - updated 28 June 2023. 2023.
2. [Available from: https://www.freemaptools.com/distance-between-uk-postcodes.htm.
3. Ritchie H. Which form of transport has the smallest carbon footprint? 2023.
4. McAlister S, McGain F, Petersen M, Story D, Charlesworth K, Ison G, et al. The carbon footprint of hospital diagnostic imaging in Australia. Lancet Reg Health West Pac. 2022;24:100459.
5. Narayanan H, Raistrick C, Tom Pierce JM, Shelton C. Carbon footprint of inhalational and total intravenous anaesthesia for paediatric anaesthesia: a modelling study. Br J Anaesth. 2022;129(2):231-43.
6. Rizan C, Lillywhite R, Reed M, Bhutta MF. The carbon footprint of products used in five common surgical operations: identifying contributing products and processes. Journal of the Royal Society of Medicine. 2023;116(6):199-213.
7. Mathers J. Green Freight Math: How to Calculate Emissions for a Truck Move 2015 [Available from: https://business.edf.org/insights/green-freight-math-how-to-calculate-emissions-for-a-truck-move/.
8. Rizan C, Bhutta MF, Reed M, Lillywhite R. The carbon footprint of waste streams in a UK hospital. Journal of Cleaner Production. 2021;286:125446.
9. Cannings E, Kenington C, Cox K. Sustainability Prize 2 Calculating the Carbon Footprint of Surgical Scrubbing. British Journal of Surgery. 2022;109(Supplement_5).
10. Improvement NEaN. C0294-letter-linen-and-laundry-15-april-2020. 2020 15 April.
11. Shahmohammadi S, Steinmann Z, Clavreul J, Hendrickx H, King H, Huijbregts MAJ. Quantifying drivers of variability in life cycle greenhouse gas emissions of consumer products—a case study on laundry washing in Europe. The International Journal of Life Cycle Assessment. 2018;23(10):1940-9.
