# Supplementary material for: Sustainability in Abdominal Wall Reconstruction: An Eco-Audit of the Abdominal Wall Reconstruction Pathway
Source: Ann Surg Open. 2025 May 15;6(2):e576. doi: 10.1097/AS9.0000000000000576 (PMC12185097; doi:10.1097/AS9.0000000000000576)
Supplement: Supplementary file 2 [file as9-6-e576-s002.pdf]

Indication for AWR	Intraoperative time in minutes (mean, IQR)	Mean inpatient stay (mean, IQR)
Hernia	211.88 (75.00)	11 (10.41)
Excess panniculus of abdomen	175.86 (37.50)	1 (0.38)
Abdominal wall injury/open abdominal wound	158.33 (98.19)	4 (3.61)
Tumour	278 (66.47)	7 (2.83)
Hypertrophic scar	134 (0)	2 (0)
Change in skin lesion	138 (0)	2 (0)

Supplementary Table 2: Difference in intraoperative time and inpatient stay based on indication for abdominal wall reconstruction. Abbreviations: AWR – Abdominal Wall Reconstruction, IQR – Interquartile Range
